# Combined VEGF and CXCR4 antagonism targets the GBM stem cell population and synergistically improves survival in an intracranial mouse model of glioblastoma

**DOI:** 10.18632/oncotarget.2443

**Published:** 2014-09-09

**Authors:** Amy Barone, Rajarshi Sengupta, Nicole M. Warrington, Erin Smith, Patrick Y. Wen, Rolf A. Brekken, Barbara Romagnoli, Garry Douglas, Eric Chevalier, Michael P. Bauer, Klaus Dembowsky, David Piwnica-Worms, Joshua B. Rubin

**Affiliations:** ^1^ Department of Pediatrics, Washington University School of Medicine, 660 South Euclid Ave, St. Louis, MO; ^2^ BRIGHT Institute, Washington University School of Medicine, 660 South Euclid Ave, St. Louis, MO; ^3^ Molecular Imaging Center, Mallinckrodt Institute of Radiology, Washington University School of Medicine, 660 South Euclid Ave, St. Louis, MO; ^4^ Center for Neuro-Oncology, Dana Farber/Brigham and Women’s Cancer Center, Brookline Ave, Boston, MA; ^5^ Division of Neuro-Oncology, Department of Neurology, Brigham and Women’s Hospital, Brookline Ave, Boston, MA; ^6^ Hamon Center for Therapeutic Oncology Research, UT Southwestern Medical Center, Harry Hines Blvd. Dallas, TX; ^7^ PolyPhor Ltd, Hegenheimermattweg 125 CH-4123 Allschwil, Switzerland; ^8^ Department of Cell Biology & Physiology, Washington University School of Medicine, 660 South Euclid Ave, St. Louis, MO; ^9^ Department of Cancer Systems Imaging, University of Texas MD Anderson Cancer Center, Holcombe Dr., Houston, TX; ^10^ Department of Anatomy and Neurobiology, Washington University School of Medicine, 660 South Euclid Ave, St. Louis, MO

**Keywords:** CXCR4, VEGF, perivascular, glioblastoma, stem cells

## Abstract

Glioblastoma recurrence involves the persistence of a subpopulation of cells with enhanced tumor-initiating capacity (TIC) that reside within the perivascular space, or niche (PVN). Anti-angiogenic therapies may prevent the formation of new PVN but have not prevented recurrence in clinical trials, suggesting they cannot abrogate TIC activity. We hypothesized that combining anti-angiogenic therapy with blockade of PVN function would have superior anti-tumor activity. We tested this hypothesis in an established intracranial xenograft model of GBM using a monoclonal antibody specific for murine and human VEGF (mcr84) and a Protein Epitope Mimetic (PEM) CXCR4 antagonist, POL5551. When doses of POL5551 were increased to overcome an mcr84-induced improvement in vascular barrier function, combinatorial therapy significantly inhibited intracranial tumor growth and improved survival. Anti-tumor activity was associated with significant changes in tumor cell proliferation and apoptosis, and a reduction in the numbers of perivascular cells expressing the TIC marker nestin. A direct effect on TICs was demonstrated for POL5551, but not mcr84, in three primary patient-derived GBM isolates. These findings indicate that targeting the structure and function of the PVN has superior anti-tumor effect and provide a strong rationale for clinical evaluation of POL5551 and Avastin in patients with GBM.

## INTRODUCTION

Glioblastoma (GBM) is the most common malignant brain tumor in adults and makes up approximately 5% of brain tumors in children [1]. Despite advances in surgery, radiation, and chemotherapy, the average survival for a patient with GBM remains just15 months from the time of diagnosis [2]. Temozolomide, an alkylating agent, is the only drug that, in combination with complete resection and radiation therapy, has been shown to improve overall survival [2, 3]. Alternate approaches, particularly those that can target the mechanisms of recurrence, are required.

Current models of GBM biology suggest that tumor recurrence depends upon the persistence of a subpopulation of cells with enhanced tumor-initiating capacity [4]. These so called tumor-initiating cells (TICs) or “tumor stem cells,” which are distinguished by their expression of stem cell markers and enhanced tumorigenicity, are purported to be a reservoir of germinal activity from which recurrent tumors derive [5, 6]. Similar to normal stem cell populations, GBM TICs reside within specialized niches [7, 8]. The perivascular space or niche (PVN) in GBM is a cellularly heterogeneous domain that is enriched with GBM TICs [9-12]. Within the PVN, functional interactions between GBM TICs microvascular endothelial cells, pericytes, astrocytes and microglia regulate TIC activity, mediate resistance to therapy and provide a conduit for invasion. Thus targeting the formation and function of the PVN has the potential to block GBM recurrence and improve cure.

The formation of new PVN depends upon angiogenesis. GBM is renowned as being among the most angiogenic cancers, and this property is highly correlated with expression and secretion of vascular endothelial growth factor (VEGF) by tumor cells [13-15]. Blocking VEGF function with Bevacizumab (Avastin^®^), a humanized monoclonal antibody directed against VEGF-A, is approved for use in several cancers, and has accelerated approval for relapsed/refractory GBM [16, 17]. Bevacizumab treatment frequently results in transient radiographic improvement and improved quality of life in patients with GBM [18]. However, despite initial response, nearly all patients progress, suggesting either resistance in angiogenic mechanisms or that tumor growth cannot be controlled by blocking angiogenesis and formation of new PVN alone[18].

Multiple cell types and signaling pathways mediate the functions of the PVN. We previously showed that endothelial cell-derived CXCL12 was required for localizing GBM cells to the PVN and also induced their growth [19]. CXCL12 and its receptor, CXCR4, are important for the functioning of multiple germinal matrices in a variety of tissues [20-22]. These functions have been successfully targeted in the clinical application of the CXCR4 antagonist AMD3100 for the mobilization of hematopoietic stem cells from their bone marrow niche and as an adjunct to chemotherapy [23-25]. In the central nervous system, the CXCL12-CXCR4 axis is critical for the function of the primary neurogenic subependymal zone as well as secondary germinal matrices in the cerebellum and hippocampus [26-28]. Disruption of this pathway by gene deletion results in significant abnormalities in hematopoiesis and brain development [28, 29].

CXCR4 expression is elevated in many brain tumor types, including GBM, where increased expression is associated with a worse prognosis [20, 30, 31]. CXCL12 is also upregulated in the brain tumor microvasculature, and blocking the CXCR4 axis with antagonists AMD3100 and AMD3465 resulted in significant anti-tumor effects in animal models [19, 20, 30-33]. In these studies both tumor cell and microvascular CXCR4 were identified as potential targets. These findings have supported the clinical evaluation of AMD3100 in the treatment of recurrent GBM, though efficacy has not been established. Here, we hypothesized that in GBM, CXCR4 antagonism works by blocking the TIC supportive effects of the PVN, and that in combination with VEGF antagonism, would exert superior anti-tumor effect by inhibiting PVN formation and function. We therefore performed a preclinical study of combined VEGF and CXCR4 antagonism in an intracranial xenograft model of GBM using the Protein Epitope Mimetic (PEM) CXCR4 antagonist POL5551 [34], together with mcr89, an anti-VEGF antibody with dual functionality against both murine and human VEGF [35].

## RESULTS

We previously demonstrated that CXCR4 antagonism could disrupt the effects of endothelial cells on GBM cells in an *in vitro* model of the PVN and block intracranial xenograft growth [19, 32, 33]. Based on these findings, we were interested in determining whether there would be an advantage of combination therapy with a VEGF antagonist. POL5551, a novel CXCR4 antagonist, was shown to produce superior bone marrow stem cell mobilization in mice compared to an established CXCR4 antagonist AMD3100 [34]. In this same study, AMD3100 also had greater dose-limiting toxicities. We hypothesized that the combination of POL5551 and mcr84 (VEGF inhibitor) would effectively target GBM PVN structure and function. We tested this hypothesis in an intracranial xenograft model of GBM using eGFP-luciferase-expressing U87 cells. U87 xenografts are highly angiogenic and prior studies using them have identified tumor cell and microvascular targets for CXCR4 antagonism [32, 36]. Thus, we used U87 xenografts to further define the cellular target(s) of CXCR4 inhibition.

**Figure 1:**
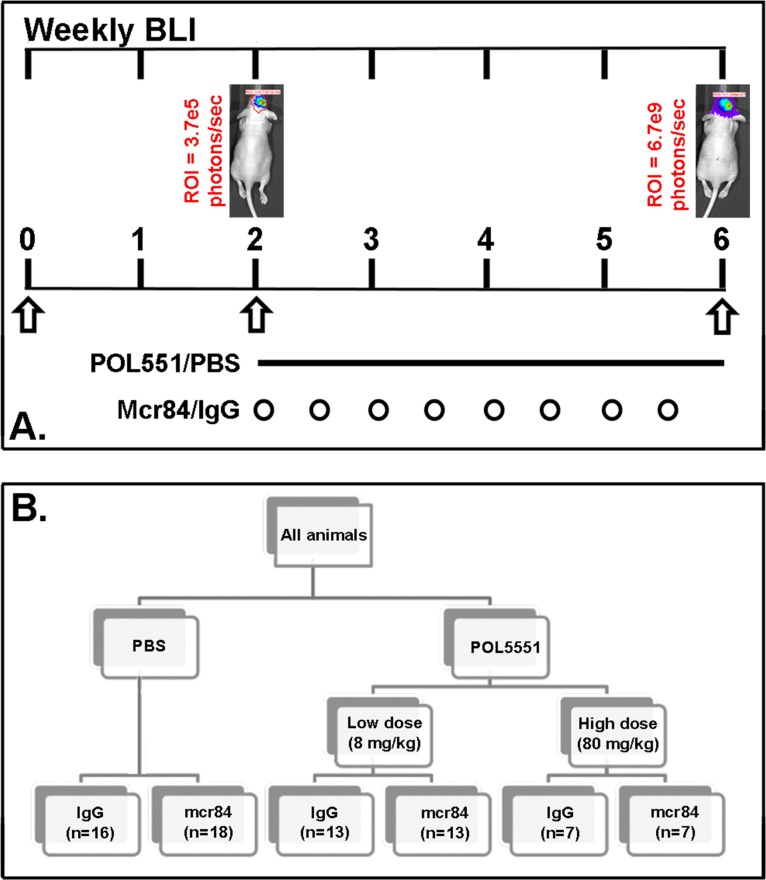
Treatment scheme (A.) Engraftment of intracranial tumors was confirmed by serial BLI over the two week post implantation period. (B) A subcutaneous osmotic pump delivered either PBS or POL5551 (low dose or high dose) continuously over 28 days. Mice received either mcr84 or vehicle IgG antibody (10 mg/kg i.p. twice weekly for 4 weeks).

**Figure 2:**
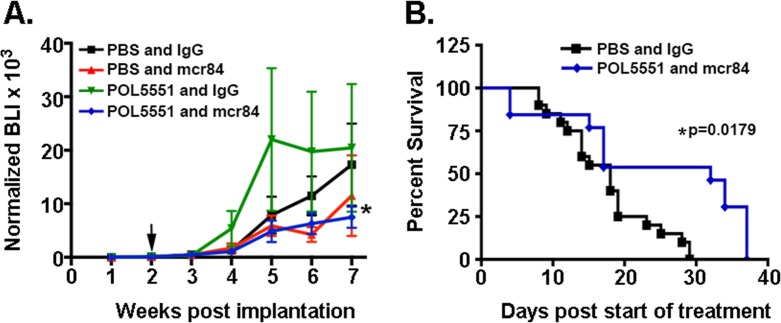
Combined mcr84 and LD-POL5551 blocks brain tumor growth and increases survival in vivo (A) Tumor growth was measured by weekly BLI. Shown are the mean and SEM for weekly BLI measurements for each treatment group (n=13-18 mice per group) normalized to fold over initial BLI. Arrows indicate the start and end of treatment. *p=0.0137 for the effect of treatment (weeks 2-6) on tumor growth (BLI). (B) LD-POL5551 in combination with mcr84 (n=13) increased median survival in comparison to vehicle controls (n=16) (p=0.0179). There were no significant improvements in survival for either of the monotherapy groups.

Animals bearing intracranial U87 xenografts that exhibited steady and equal growth over the two-week post-impantation period were randomly assigned to one of four different treatment groups: PBS and IgG (Control), low dose POL5551 (LD-POL5551, 8mg/kg/day) and IgG, PBS and mcr84 (10mg/kg twice weekly), LD-POL5551 and mcr84 (Figure 1). Mice were treated for a total of four weeks, and during the treatment period (week 2 to week 6) mcr84 alone or mcr84 in combination with LD-POL5551, significantly inhibited intracranial tumor growth to an equivalent level as measured by weekly BLI (Figure 2A). Tumor growth persisted after the cessation of treatment at 6 weeks. While the addition of POL5551 to mcr84 did not enhance the inhibition of tumor growth, analysis of survival indicated there was a benefit to the combination. Median survival was similar between control (18 days), mice treated with LD-POL5551 alone (17 days) or mice treated with mcr84 alone (18 days). However, mice treated with both LD-POL5551 and mcr84 exhibited significantly longer median survival (32 days) compared to control mice (p=0.0179) (Figure 2B). These results indicated possible synergy between the drugs.

**Figure 3:**
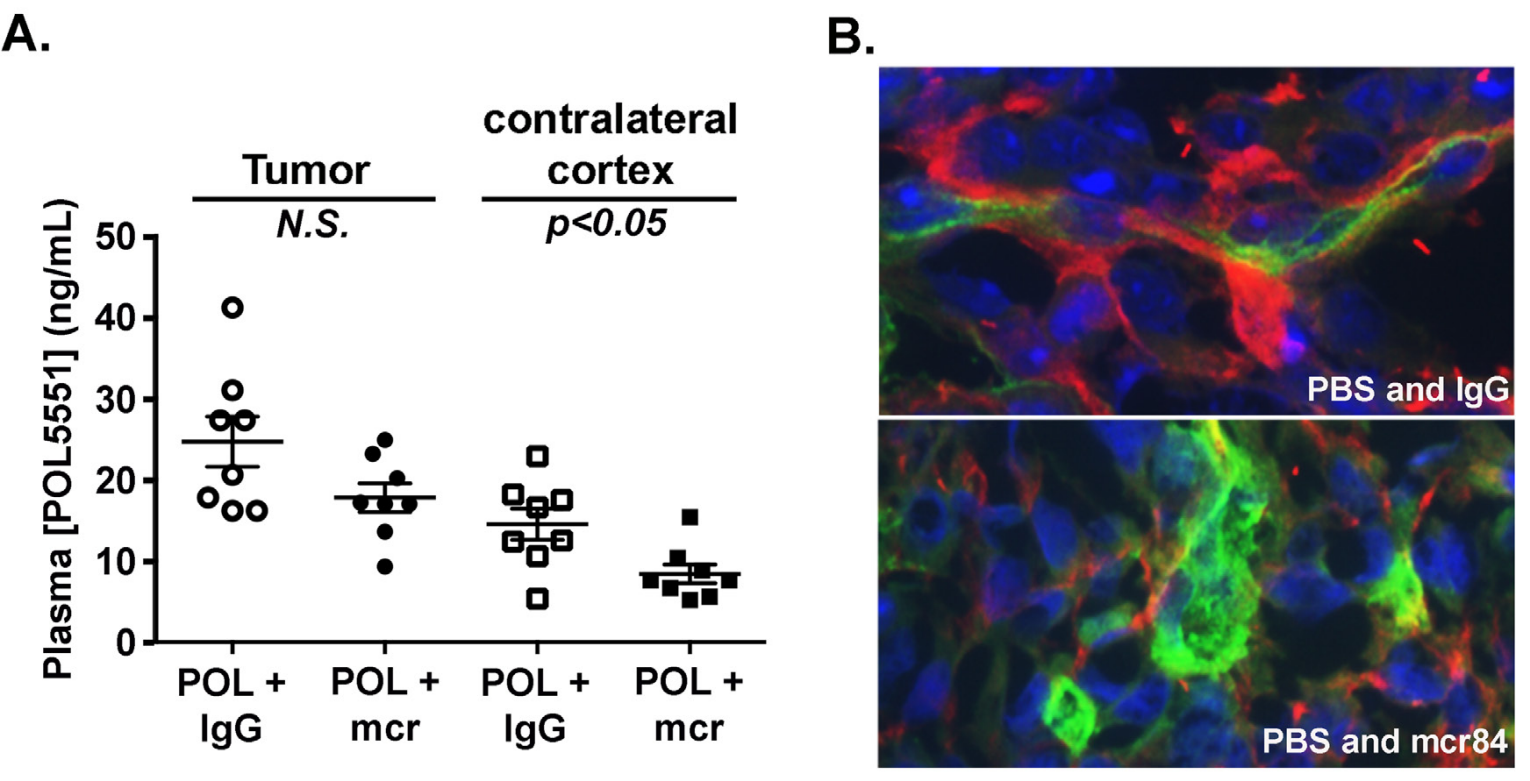
VEGF blockade with mcr84 enhances vascular barrier functions (A) POL5551 concentrations in tumor tissue and non-tumor tissue from the contralateral cortex of xenograft bearing mice were determined by LC-MS/MS. POL5551 concentration was higher in tumor tissue versus non-tumor tissue. In both the tumor and non-tumor tissue, POL5551 concentration was decreased in the presence of mcr84. Bars represent the mean±SEM values. (B) mcr84 treatment markedly reduced the amount of albumin (red) detectable within the perivascular space of tumor sections compared to mice treated with vehicle control. Vascular endothelial cells were identified by endoglin staining (green) and nuclei are counterstained in blue with DAPI.

To further investigate interactions between POL5551 and mcr84, we measured compound levels in blood plasma, tumor–bearing cortex, and contralateral (non-tumor bearing) cortex. Consistent with an intact blood brain barrier (BBB) limiting brain permeation of POL5551, mean concentrations of POL5551 in normal brain tissue were 13-fold lower than in plasma (not shown). Compared to contralateral non-tumor bearing cortex, mean concentrations of POL5551 in the tumor bed were 1.7-fold higher (Figure 3A), indicating disruption of normal BBB function. Importantly, treatment with mcr84 lowered the mean concentrations of POL5551 in tumor tissue and normal cortex by 28% and 42%, respectively. These findings suggested that, like Avastin®, mcr84 might normalize and improve barrier function within the tumor vasculature [37, 38]. To determine whether the barrier effects of mcr84 could be extrapolated to other molecules, we examined the extravasation of albumin in tissue sections from control and mcr84 treated animals. Throughout tumor sections from mice treated with PBS and IgG control, we found albumin within the perivascular space (Figure 3B). mcr84 treatment markedly reduced the amount of albumin observed within the perivascular space indicating that VEGF antagonism results in improved BBB function.

**Figure 4:**
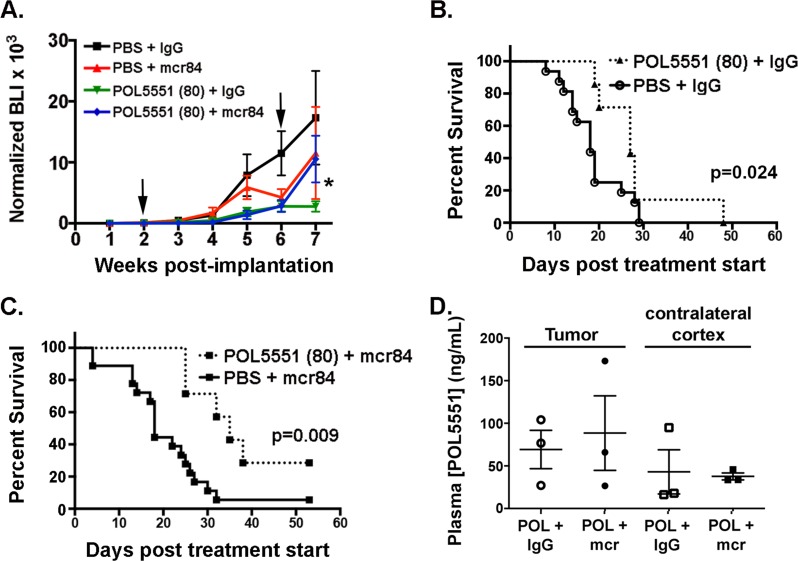
HD-POL5551 blocks brain tumor growth and increases survival *in vivo* (A) Tumor growth was measured by weekly bioluminescence imaging. Shown are the mean and SEM for weekly BLI measurements each treatment group (n=7-18 mice per group). Arrows indicate the start and end of treatment. *p=0.0129 for the effect of treatment (weeks 2-6) on tumor growth (BLI). (B,C) HD-POL5551 alone (n=7) and in combination with mcr84 (n=7) increased mean survival in comparison to vehicle controls (n=16) (p=0.014 and p=0.009 respectively). (D) POL5551 concentrations in tumor tissue and non-tumor tissue from the contralateral cortex of xenograft bearing mice were determined by LC-MS/MS. POL5551 concentration was higher in tumor tissue versus non-tumor tissue. Bars represent the mean±SEM values. There was no significant decrease in tumor levels upon combination with mcr84.

Decreased permeation of POL5551 in the setting of VEGF antagonism might reduce potential therapeutic efficacy. To test this hypothesis, we increased the POL5551 dose ten-fold and sought to determine whether this could overcome the permeation barrier and exert a superior anti-tumor effect. Four weeks of 80 mg/kg/day POL5551 (high dose (HD)) alone or in combination with mcr84 significantly inhibited intracranial xenograft growth (Figure 4A). Interestingly, upon cessation of therapy at 6 weeks, tumor growth appeared to rebound in the mcr84-containing regimens.

Consistent with the inhibition of tumor growth, there was a significant increase in median survival from 18 to 27 days in animals treated with the HD-POL5551 alone compared to control (p=0.024 (Figure 4B). The addition of mcr84 to HD-POL5551 further increased median survival to 35 days, which was a significant improvement compared to mcr84 (18 days) alone (p=0.009) (Figure 4C). The difference in median survival between mcr84 combined with low dose (32 days) and high dose (35 days) POL5551 did not reach statistical significance but there was an apparent difference in overall survival. In the groups treated with the mcr84 and POL551 combination, there were no survivors by day 37 when LD-POL551 was used, whereas 30% survived beyond this timepoint HD-POL5551was used.

To determine whether the improved survival was associated with increased tumor tissue POL5551, we confirmed compound concentrations by mass spectrometry. At the higher dose of POL5551, mean concentrations were increased approximately 3-fold in tumor and non-tumor bearing (contralateral) cortex compared to animals receiving LD-POL5551 (Figure 4D). Importantly, tumor POL5551 concentrations following HD-POL5551 were not significantly decreased upon combination with mcr84.

**Figure 5:**
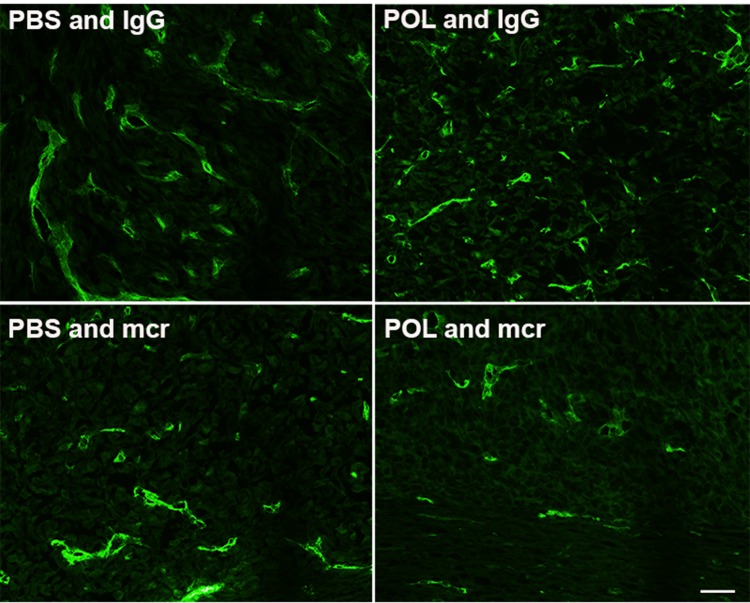
Treatment with mcr84 reduces endoglin positivity Tumor tissue from each of the treatment groups was evaluated for endoglin positive endothelial cells (green). mcr84 alone or in combination with HD-POL5551 reduced the amount of endoglin positivity. HD-POL5551 had no effect on endoglin positivity. Scale bar equals 20 microns.

**Figure 6:**
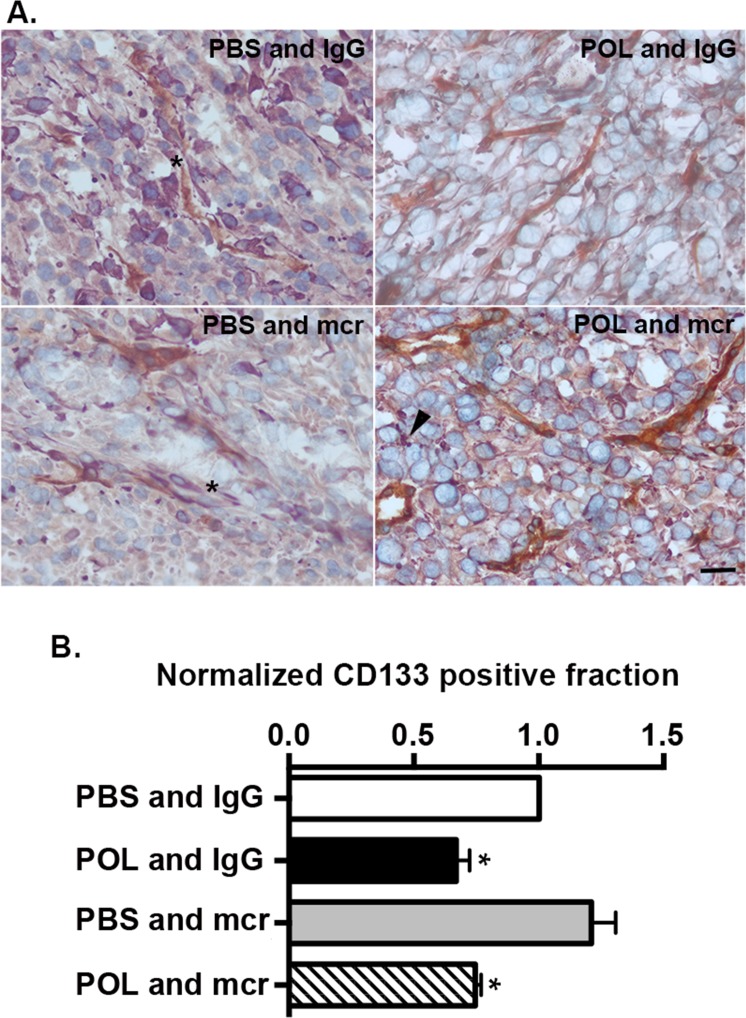
HD-POL5551 reduces the GBM stem cell fraction *in vivo* and *in vitro* (A) Tumor tissue from each of the treatment groups was evaluated for endoglin (brown) and nestin (purple) expression. Nuclei were counterstained with methyl green. In the PBS and IgG and PBS and mcr84 groups, numerous nestin positive cells can be seen within the perivascular space (asterisks). In the HD-POL5551 and IgG and the HD-POL5551 and mcr84 groups, there are few to no nestin positive cells within the perivascular space. Nestin positivity is evident as punctate staining (arrowhead) rather than the usual diffuse cytoplasmic staining. Scale bar equals 20 microns. (B) CD133 fraction was measured by MACS in three different low passage isolates from GBM patients. Presented are the means and SEM of these measurements. * Indicates P<0.05 as determined by two-tailed t-test.

To evaluate the mechanism of HD-POL5551 anti-tumor effects we performed tissue based analyses to examine tumor morphology, and tumor cell proliferation and apoptosis. None of the treatments altered the characteristic non-infiltrative pattern of U87 growth (data not shown). As anticipated, treatment with mcr84 alone or in combination with HD-POL5551 was associated with a decrease in endoglin positivity (Figure 5) which is indicative of decreased endothelial cell numbers. Proliferation was measured with Ki67 immunofluorescence and apoptosis was measured by TUNEL staining. For both measurements, positivity was determined in a blinded fashion in multiple high-powered fields from three separate subjects from each treatment group. All treatment regimens were associated with significant decreases in proliferation and increases in apoptosis compared to vehicle-treated controls as determined by two-tailed Fisher’s exact test (Table 1). In addition, treatment with HD-POL5551 alone or in combination with mcr84 had a widespread effect on the shape and size of tumor cell nuclei and appeared to reduce the numbers of nestin-positive perivascular cells (Figure 6A, asterisks). Nestin is an intermediate filament expressed by normal neural and brain tumor stem cells [39, 40]. It typically fills the cytoplasm of positive cells as seen in brains from PBS and IgG or PBS and mcr84 treated animals. Interestingly, nestin positivity in tumors treated with HD-POL5551 was reduced to puncta (Figure 6A, arrowheads).

**Table 1:** Drug effects on proliferation and apoptosis in U87 tumors

	Ki67				TUNEL			
Treatment Group	Fraction^1^	Pos	Neg	P-value^2^	Fraction	Pos	Neg	P-value
PBS and IgG	0.28	567	1477		0.12	242	1793	
PBS and mcr	0.24	443	1440	0.0027	0.14	297	1866	0.0794
POL and IgG	0.21	418	1593	0.0001	0.25	290	873	0.0001
POL and mcr	0.21	377	1392	0.0001	0.17	371	1792	0.0001

1 Fraction of Ki67 positive or TUNEL positive cells was calculated as #positive cells/(#positive cells + #negative cells) in multiple high-powered fields from three brains from each treatment group.

2 P values were calculated using a two-tailed Fisher’s exact test to compare treatment groups to control (PBS and IgG).

HD-POL5551 overcame the permeation barrier of VEGF antagonism and was associated with a superior anti-tumor effect and changes in tumor cell nuclear shape, proliferation and apoptosis compared to PBS controls (PBS+IgG and PBS+mcr84) (Table 1, Figure 6). These findings support the literature that states CXCR4 antagonist targets are not limited to the vascular endothelium but include tumor cell targets as well [19, 32]. Taken together with other reports describing a role for CXCR4 in the regulation of neural stem cell function [41, 42] our data showing that POL5551 reduced the numbers of nestin positive perivascular cells, led us to hypothesize that POL5551 might have direct effects on tumor stem cells. To test this, and improve the translational relevance of these studies, we investigated the direct effects of POL5551 on three primary GBM isolates.

The stem cell or TIC fraction of primary GBM isolates was measured as the CD133 positive fraction in tumorsphere cultures. CD133 positive cells in three low passage GBM preparations (CDI-2, CDI-5, B18) were isolated by MACs with antibody directed against CD133. To measure the effect of POL5551 on the TIC fraction, cells were treated with TSM with IgG (10 μg/mL) as vehicle control, POL5551 (1 μg/mL) and IgG, PBS and mcr84 (10 μg/mL), or POL5551 and mcr84 together. After treatment, the CD133 fraction was measured again and normalized to vehicle. mcr84 alone had no effect on the CD133 positive fraction. In contrast, POL5551 alone or in combination with mcr84 reduced the CD133 positive fraction to 70% of control values (Figure 6B). Together, these data indicate that CXCR4 antagonism reduces the GBM TIC fraction through direct action, requiring drug permeation into tumor tissue.

## DISCUSSION

Despite decades of research, GBM remains among the most devastating malignancies. Targeting both the function and formation of the PVN could be important for preventing tumor growth, invasion, and metastasis. In this study, we showed that the combination of a CXCR4 antagonist, POL5551, and a VEGF inhibitor, mcr84, can increase median overall survival in GBM xenografts compared to treatment with either drug as monotherapy.

CXCR4 and VEGF are two of the few validated targets for directed GBM therapy. Bevacizumab, a humanized monoclonal antibody against VEGF-A, disrupts the formation of tumor growth by inhibiting angiogenesis; however, it does not improve cure. CXCR4 expression is well known to be increased in many cancers, including GBM, and is associated with poor prognosis [43]. We showed that AMD3100 or AMD3465, related CXCR4 antagonists, inhibit the growth of intracranial GBM xenograft models by decreased proliferation and increased apoptosis of tumor cells [32, 33]. The CXCR4/CXCL12 pathway has also been shown to be involved in GBM invasiveness [30, 31]. AMD3100 is currently being evaluated in clinical trial in combination with bevacizumab for patients with high-grade gliomas (NCT01339039). In prior studies, sustained AMD3100 treatment had significant side effects, including unexplained cardiotoxity [44]. By targeting formation and function of the tumor PVN, we hoped to decrease tumor growth and invasiveness synergistically and increase survival. POL5551 is thought to have less toxicity and has been shown to have superior ability to mobilize bone marrow stem cells in a mouse xenograft model [34]. The improved safety and efficacy of POL5551 over other CXCR4 antagonists makes it an attractive therapeutic candidate.

POL5551 was first studied in xenografts for the use of stem cell mobilization at a dose of 8 mg/kg/day, prompting our initial dose for this experiment. We saw a significant increase in survival of mice treated with the combination of LD-POL5551 and mcr84 compared to vehicle or either drug alone, suggesting possible synergy. Measurement of tissue POL5551 concentrations revealed that in the presence of mcr84, POL5551 concentrations were decreased in normal brain as well as tumor tissue, suggesting mcr84 decreased blood vessel permeability and subsequent permeation of POL5551. VEGF was originally known as vascular permeability factor (VPF) because of its influence on the permeability of blood vessels [13]. Our results are consistent with several recent studies that showed VEGF antagonists, including bevacizumab, negatively affect concomitant drug penetration into tumor tissue [45-47]. For this reason, we increased the dose of POL5551 by 10-fold which significantly increased POL5551 concentrations in plasma, tumor, and normal brain and significantly increased median survival.

The association of higher POL5551 concentrations in tumor tissue with increased survival suggests that a critical target for POL5551 action is within the tumor parenchyma. This conclusion is supported by the apparent POL5551-induced decrease of nestin positive stem cells *in vivo*, and additionally by the reduction of the CD133 positive cell fraction in three independent primary GBM isolates by POL5551 in our *in vitro* studies. Together, these studies strongly support the clinical evaluation of combined VEGF and CXCR4 antagonism, but with increased doses of the CXCR4 antagonist to overcome the improved vascular barrier function that results from effective VEGF antagonism.

## MATERIALS AND METHODS

Animals were used in accordance with an Animal Studies Protocol (# 20120174) approved by the Animal Studies Committee of the Washington University School of Medicine per the recommendations of the Guide for the Care and Use of Laboratory Animals (National Institutes of Health).

### Human Studies

Primary human GBM specimens for culture were obtained and utilized in accordance with a Washington University Institutional Review Board (IRB)-approved Human Studies Protocol (#201102299).

### Chemicals and other reagents

POL5551 was supplied by Polyphor and mcr84 was obtained from Affitech AS and purified in house by Protein A chromatography. All tissue culture reagents and media were obtained from Invitrogen unless otherwise indicated.

### Tumor cell lines

U87 cells were obtained from the American Type Culture Collection (Manassas, VA) and validated by them. Viral particles were produced by the Viral Vectors Core Facility of The Hope Center for Neurological Diseases at Washington University School of Medicine; expression vectors used in viral production contained a transgene for green fluorescent protein-firefly luciferase (GFP). GFP expressing cells were sorted and collected by high-speed fluorescence-activated cell sorting (MoFlo High-Performance Cell Sorter; DAKO). The eGFP-luciferase expressing U87 cells are simply referred to as U87 cells in this study. Cells were cultured in alpha-MEM supplemented with 10% FBS (Biomedia, Foster City, CA) at 37^°^ with 5% CO_2_.

### Primary Human GBM cells

Fresh brain tumor resection material from pediatric (CDI) or adult (B18) glioblastoma patients was obtained according to a Washington University School of Medicine IRB approved human studies protocol. Resection material was minced into small pieces using sterile scalpels and dissociated in Accutase at 37°C. Single cells were obtained and cultured in tumor sphere media (TSM), which contains Neurobasal-A media (Gibco) supplemented with Glutamax (Gibco), 20 ng/mL epithelial growth factor (EGF, Sigma), 20 ng/mL basic fibroblastic growth factor (bFGF, Chemicon), 20 ng/mL leukemia inhibitory factor (LIF, Chemicon, 1 × N2 Supplement (Gibco), 1 × B-27 Serum-Free Supplement (Gibco), and heparin (20 ug/mL, Sigma). Cells were initially plated on tissue-culture coated plates overnight to allow non-stem-like cells to attach, and the non-adherent stem-like cells were transferred to extracellular matrix protein (ECM)-coated tissue culture plates prepared by coating with 10% ECM (Sigma) in Hanks Buffered Saline Solution (HBSS) and washed three times in HBSS. Thereafter, GBM cells were maintained in adherent culture on ECM-coated plates in TSM media, which was changed every 2 to 3 days.

### *In vitro* drug treatments

Primary tumor cells were thawed, counted and equal numbers of cells were plated in ultra-low adherent 6 well plates in 2mls of regular TSM supplemented with 10 μg/mL IgG (vehicle), or mcr84 alone (10μg/mL), POL5551 (1μg/mL) and IgG, or POL5551 and mcr84 together. The CD133+ cell fractions were measured 72 hrs later using CD133 conjugated microbeads (Miltenyi Biotech) using manufacturer’s protocol. Briefly, tumorspheres were dissociated using trypsin and collagenase and incubated with FC blocking buffer and CD133 microbeads for 30 min. The cells were washed in MACS buffer and loaded on to the MACS columns. The columns was washed three times, detached from the magnet and cells were eluted in TSM. Cell number was counted using trypan blue exclusion.

### Generation of Intracranial xenografts

Intracranial xenografts were generated as previously described [32, 33]. In brief, 50,000 U87 cells in 7.5uL of PBS were injected into homozygous NCR nude mice (Taconic Farms, Germantown, NY) through a 27-gauge needle over 2 minutes at 2mm lateral and posterior to the bregma and 3mm below the dura.

### Bioluminescence imaging

Bioluminescence imaging (BLI) was performed as previously described [32, 33, 48]. Briefly, after injection with 150 ug/g D-luciferin (Biosynth) in PBS (i.p.), isoflurane-anesthetized mice were imaged with a charge-coupled device camera-based BLI system (IVIS 50; Xenogen Corp., Alameda, CA; exposure time= 1-60 s, binning=8, field of view=12, f/stop=1, open filter). Signals were displayed as photons/s/cm2. Regions of interest were defined manually at 95% of the maximum pixel output using Living Image and IgorPro Software (v 2.50) and data were expressed as total photon flux (photons per second). Generally, the first mouse images were obtained 3-5 days following intracranial inoculation, then weekly. Data were analyzed and plotted as the ratio of bioluminescence on a given treatment day over bioluminescence on the first day.

### *In vivo* Drug Treatment

Mice were imaged two times after implantation of cells to identify those with equivalent tumor growth rates. Two weeks after tumor cell implantation, cohorts of mice with approximately equivalent tumor bioluminescence were divided into control and treatment groups (5-6 mice per group per cohort). POL5551 was delivered as a continuous subcutaneous infusion. An osmotic pump (Alzet® model 2004, 28 day delivery) was inserted subcutaneously into each mouse and contained either PBS or POL5551 (8mg/kg or 80mg/kg). Prior to insertion, mice were anesthetized with ketamine hydrochloride at 150 mg/kg and xylazine at 12 mg/kg (Phoenix Pharmaceuticals, St Joseph, MO) via i.p. injection. The incision was closed with Vetbond (3M, St. Paul, MN). mcr84 (10mg/kg [35]) or IgG antibody were injected i.p. twice weekly for 4 weeks.

Mice were euthanized per protocol if they exhibited weight loss of greater than 15% or displayed neurological symptoms. Blood samples were collected for plasma analysis. All mice were perfused with PBS; a subset was fixed with 4% paraformaldehyde. If fixed, brains were stored in paraformaldehyde for 24 hours, then 15% sucrose for 24 hours, then 30%-sucrose for 24 hours. Brains were embedded in OTC and stored at −80′C. 10-12um sections were made for immunostaining. The brains of mice perfused only with PBS were frozen in liquid nitrogen then stored at −80′C. Tumor-bearing cortex and non-tumor-bearing (contralateral) cortex from these brains were further processed for determination of POL5551 concentration.

### Immunostaining

Sections (10um) were processed and analyzed as described^37^. For endoglin/nestin colabeling studies, tissue sections were permeabilized with 0.025% Triton-X-100 and endogenous peroxidase, and nonspecific binding sites were blocked as previously described [49]. Primary antibody concentrations were as follows: Endoglin (R&D systems AF1320, Goat, 1:200 dilution) and Nestin (Sigma N5413, rabbit, 1:200 dilution). Staining was detected using biotin-conjugated secondary antibodies augmented by streptavidin-horseradish peroxidase. Endoglin and Nestin were visualized using 3,3′-diaminobenzidine and the Vector VIP Peroxidase Substrate kit (DAKO), respectively. Sections were counterstained with methyl green. Control sections were processed identically in the absence of primary antibody.

Endoglin/albumin colabeling, and TUNEL and Ki67 staining was detected by immunofluorescence using the secondary AlexaFluor 555–conjugated and AlexaFluor 488–conjugated secondary antibodies at a concentration of 1:1200 (Molecular Probes) for 90 minutes. Primary antibodies were as follows: albumin (Abcam Cat#: ab2406, rabbit polyclonal, 1:1000 dilution), Endoglin (R&D systems AF1320, Goat, 1:200 dilution), and Ki67 (Thermo Scientific RM-9106-S0, Rabbit monoclonal, 1:500 dilution.) Nuclei were counterstained with 4′,6-diamidino-2-phenylindole (DAPI). Control sections were processed identically in the absence of primary antibody.

TUNEL staining was performed using the TACS 2 TdT In situ Apoptosis Detection Kit (Trevigen Cat# 4810-30-CK) per manufacturer’s instructions. Briefly, tissue sections were treated with 5ug/ml proteinase K, followed by incubation in labeling reaction mix (Tdt dNTP Mix, TdT Enzyme, Mn^2+^, and 1XTdT labeling Buffer, for 60 minutes at 37°C in humidified chamber, followed by incubation with the 1X Tdt Stop Buffer. Sections were washed in 1X PBS and incubated in AlexaFluor 568–conjugated secondary antibody (1:1500) (Molecular Probes) for 90 minutes. Nuclei were counterstained with 4′,6-diamidino-2-phenylindole (DAPI). Control sections were processed identically in the absence of Tdt Enzyme.

### POL5551 concentration

Pretreatment plasma samples and plasma, brain and tumor tissue from the time of death were obtained and stored at −20C. Samples were processed by Polyphor by mass spectrometry analysis. Concentrations of POL5551 in plasma and brain were determined using a high pressure liquid chromatography coupled to mass spectrometry detection (LC-MS/MS analytical method). Briefly, after addition of an internal standard, plasma and brain homogenate samples (aliquot of 50 μL) were extracted with acetonitrile (acidified with formic acid). Supernatants were evaporated to dryness under a stream of nitrogen, and reconstituted in H_2_O/ACN, 95/5, v/v, +0.2% formic acid. Extracts were then analyzed by reverse-phase chromatography (Acquity BEH C18 column, 100 × 2.1 mm, 1.7 μm column), using an acidified water/acetonitrile gradient elution (UPLC, Waters). The detection and quantification was performed by mass spectrometry, with electrospray interface in positive mode and selective fragmentation of analytes (AB Sciex 4000 Q Trap mass spectrometer). Standards, Quality Controls and samples were extracted and assayed in the same manner.

### Statistical analysis

Data were analyzed using GraphPad Prism version 4.00 (GraphPad Software). Kaplan-Meier survival curves were analyzed using pairwise log-rank tests. Statistical differences in tumor growth curves (measured by bioluminescence) were analyzed using two-way ANOVA. The effect of drug treatments on proliferation and apoptosis were evaluated by two-tailed Fisher’s exact test. The effects of drug treatments on stem cell fraction were evaluated by two-tailed t-test.
